# A Rare Case of Fat Embolism Syndrome Masquerading as COVID-19 Pneumonia

**DOI:** 10.7759/cureus.23384

**Published:** 2022-03-22

**Authors:** Zaheer Faizi, Daniel W Kim, Ammar Humayun, Amber Batool, Ashanthi Ratnasekera

**Affiliations:** 1 General Surgery, Crozer-Chester Medical Center, Upland, USA; 2 Surgery, Crozer-Chester Medical Center, Upland, USA; 3 Surgery, Christiana Care, Wilmington, USA

**Keywords:** tibia, long bone fracture, polytrauma, ards, fat embolus, covid-19

## Abstract

In the wake of the novel coronavirus disease 2019 (COVID-19) pandemic and its associated mortality and virulence, a high clinical suspicion must be maintained for all patients presenting with respiratory failure. However, there are well-known disease processes that may have a similar presentation. We present a case of a 25-year-old male who suffered a right tibia fracture after a motor vehicle collision. He had acute hypoxic respiratory failure within 24 hours of admission, requiring mechanical ventilation. His condition significantly improved with airway pressure release mode of ventilation and proning. Although his chest CT demonstrated characteristic findings of COVID-19, he subsequently tested negative. The differential included aspiration pneumonia and fat embolism syndrome from the lower extremity fracture. Fat embolism syndrome can very closely mimic COVID-19. The rapid onset and improvement of symptoms coupled with serial negative COVID-19 testing may aid in the diagnosis.

## Introduction

The coronavirus disease 2019 (COVID-19) pandemic has led clinicians to have high suspicion as a differential diagnosis for hypoxic respiratory failure. Its rapid diagnosis is critical for disease containment. Findings suggestive of COVID-19 would move its diagnosis to the forefront in the differential diagnosis. Fat embolism syndrome is a well-known disease process occurring in polytrauma patients and patients with long bone fractures [1]. Here, we present a case of a trauma patient who presented with signs similar to COVID-19 but was eventually suspected to have fat embolism syndrome.

## Case presentation

**Figure 1:**
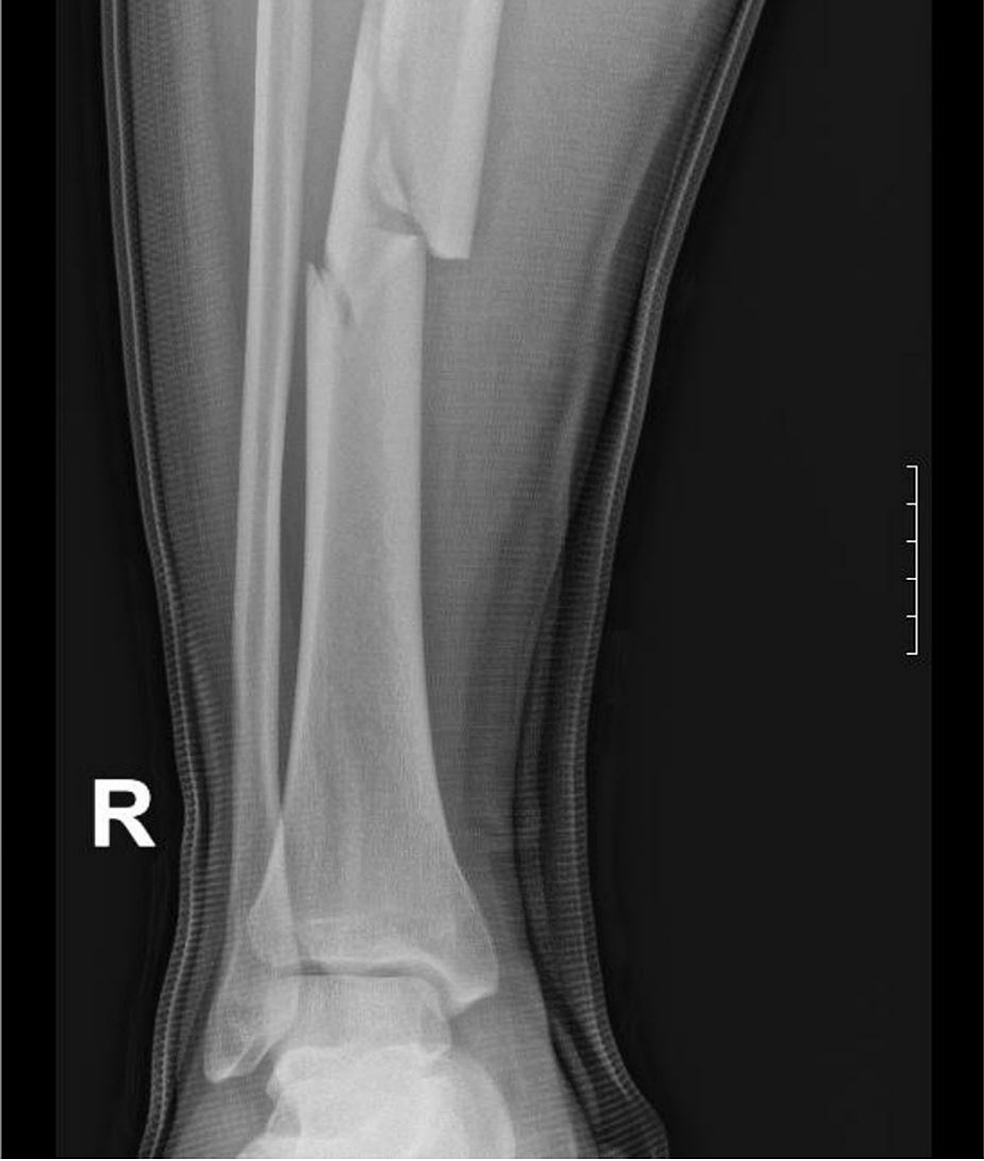
Comminuted fracture of the right tibia.

**Figure 2:**
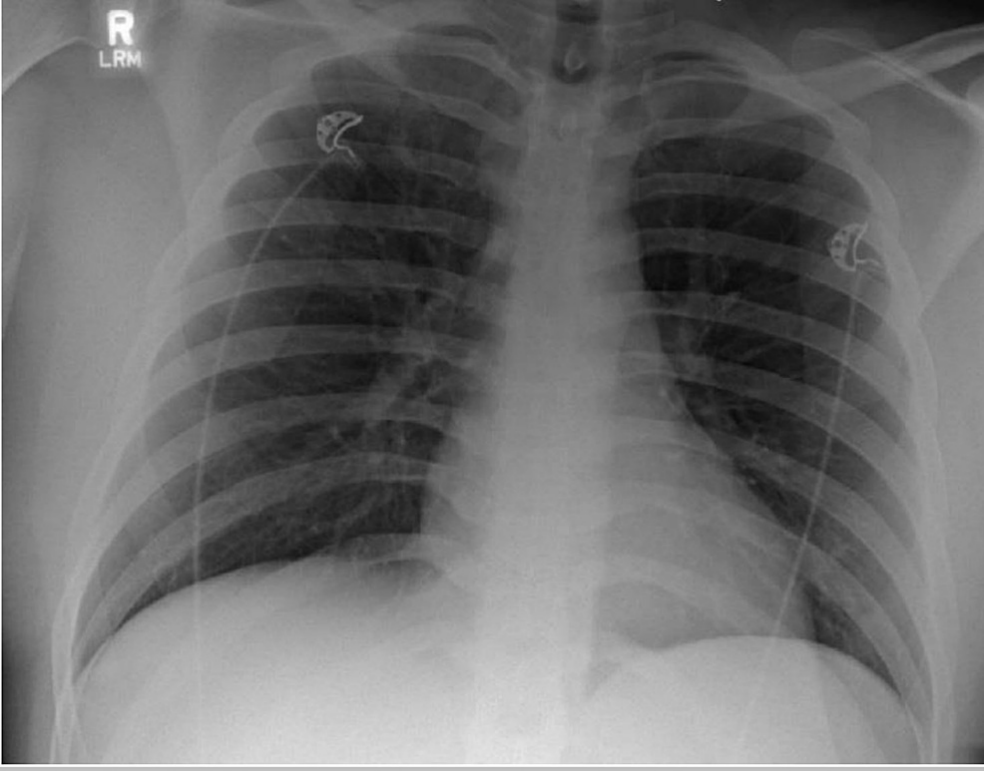
Initial chest X-ray upon presentation.

A 25-year-old-male with no past medical history presented to our level 2 trauma center with a chief complaint of right leg pain. He was a restrained driver in a 40-mile per hour motor vehicle collision with airbag deployment. There was no reported loss of consciousness and he was able to self-extricate on the scene. On the Advanced Trauma Life Support survey, there were no signs of respiratory distress. He exhibited tenderness to the right tibia with no gross deformity. His trauma work-up consisted of a CT of the head, cervical spine, chest, abdomen, and pelvis which were all negative. The patient suffered a right tibia fracture confirmed by X-ray (Figure 1). His initial chest X-ray (Figure 2) and CT chest showed no acute pulmonary process.

**Figure 3:**
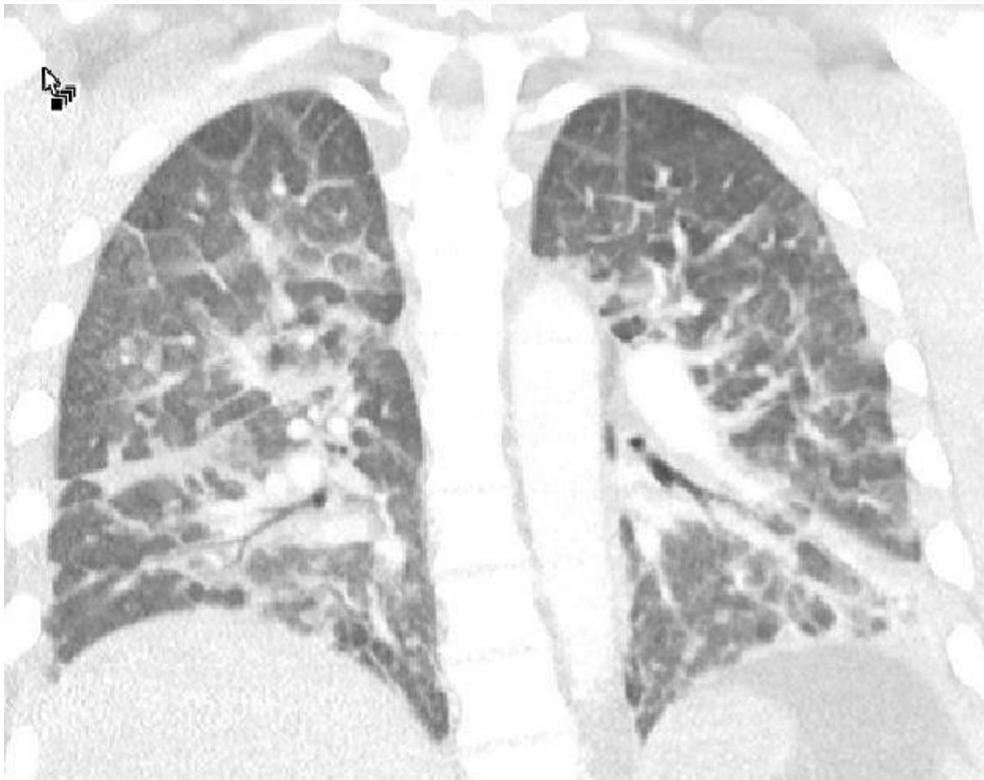
Initial CT angiography chest demonstrating multifocal lung infiltrates.

**Figure 4:**
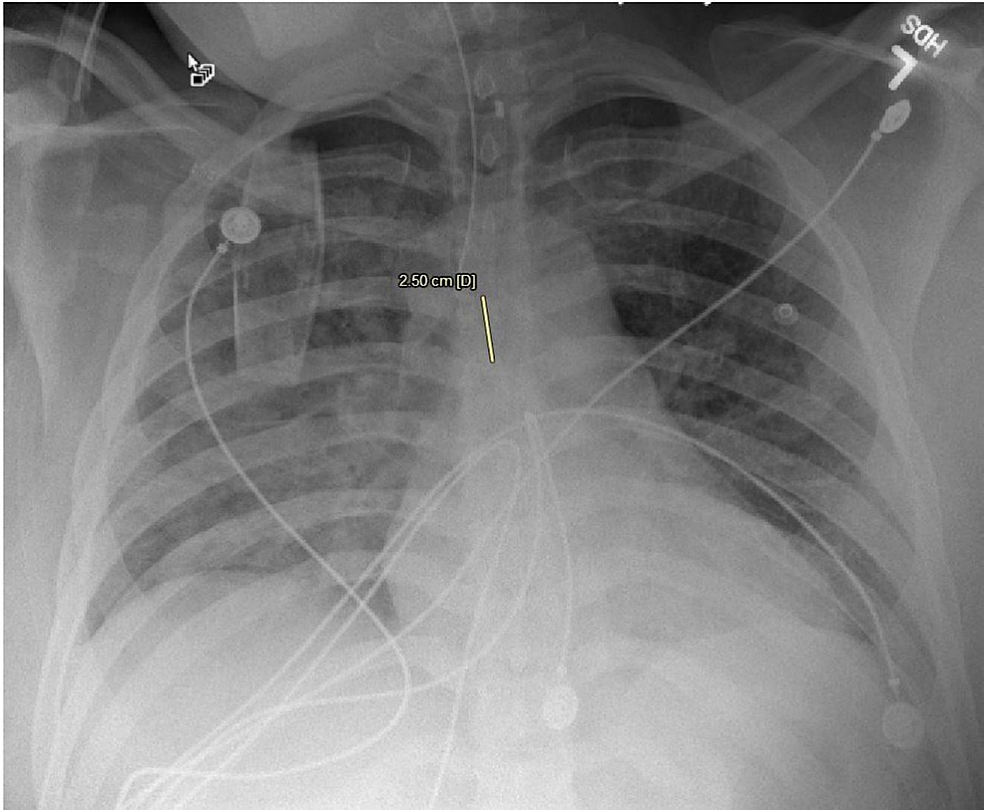
Post-intubation chest X-ray.

Within 24 h of admission, the patient was febrile to 101.3°F, tachycardic and hypoxic, saturating 93% on 3 L nasal cannula. Overnight, his fever curve increased to 102.9°F, he had worsening hypoxia with the need for 100% FiO2 nonrebreather mask. An arterial blood gas (ABG) at this time showed a PaO2 of 61 mmHg. A CT angiogram of the chest (Figure 3) was negative for pulmonary embolism but showed septal thickening within upper and lower lobes, patchy ground-glass opacities, and airspace consolidation in lower and upper lobes. A COVID-19 reverse transcriptase-polymerase chain reaction (RT-PCR) (C-PCR) test was sent immediately. He was subsequently intubated for hypoxic respiratory failure. Following mechanical ventilation on 100% FiO2, his ABG demonstrated a PaO2 of 70 mmHg. His immediate post intubation chest X-ray (Figure 4) showed new diffuse patchy alveolar opacities in both lungs more prominent on the right than left. Due to high suspicion of COVID-19, the patient was contained in a COVID intensive care unit and was then proned. Five hours post intubation and proning a repeat ABG showed a marked improvement of his PaO2 to 353 mmHg.

Initial labs sent along with the original C-PCR showed a D-Dimer of >4.0 [normal (N) (0.00-0.50), a C-reactive protein of 228 (N: <5.0), ferritin of 344 (N: 22-336), and a procalcitonin of 0.53 (N: <0.50)]. Initial blood and sputum cultures were negative. There was a 47% drop in platelets over the first four days from 324 to 171. His hemoglobin decreased from 13.8 to 9.1 on hospital day (HD) four.

Our patient’s original C-PCR test on HD 2 and HD 4 returned negative. Subsequently, a COVID-19 serology (C-Ag) was sent on HD 7 and returned negative. The patient was then transferred to a non-COVID unit with supportive care. He received a seven-day course of piperacillin/tazobactam, which was initiated on HD 2; total parenteral nutrition was initiated due to a prolonged ileus and was later transitioned to tube feeds when the ileus was resolved. His initial proning schedule was 16 h prone and 8 h supine and continued from HD 2 until HD 5. From HD 6 to HD 7 he was prone for 8 h and supine for 16 h. Proning was stopped on HD 8. Concurrently while intubated, our patient has also received chest physiotherapy via a percussion vest every four hours along with standing dual nebulizer and inhaled acetylcysteine therapy. He underwent a right tibia open reduction and intramedullary nail fixation on HD 10. He was extubated on HD 13. At this point, the patient was severely deconditioned and exhibited critical illness myopathy. On HD 17 he began to have intermittent fevers and a repeat C-PCR was sent and was negative.

## Discussion

The patient presented to our institution during a surge of COVID-19 in our geographical region. The rapid diagnosis and containment of diseases in the hospital is critical while treating patients during COVID-19 pandemic. Acute respiratory failure due to other causes must be part of the differential diagnosis. However, as in this case though there were similar presentation characteristics such as peripheral and bilateral ground glass and/or consolidative opacities, the patient’s underlying pathology was not in fact COVID-19. Furthermore, his acute desaturation after presentation without significant co-morbidities and blunt chest trauma lead to high suspicion of COVID-19. The known rate of false-negative PCR tests can also lead the clinician to continue to take COVID-19 precautions with patients with acute respiratory failure. In a retrospective study by Long et al., six initial cases of COVID-19 pneumonia tested negative (RT-PCR) with three more patients testing positive in the second test and the remaining three on the third RT-PCR. In that same subset of 36 patients, only one did not have CT findings, leading to a sensitivity of 97.2% for CT and initial sensitivity of 83.3% of RT-PCR [2]. Further, inflammatory markers that are consistent with COVID-19 were also markedly elevated in the patient described above further leading to an inconsistent diagnosis. An initial hypothesis of overwhelming incitation of inflammatory mediators caused by trauma exacerbating an underlying COVID-19 infection was made. However, our subsequent COVID-19 tests were negative. Although high false-negative rates of COVID-19 PCR tests are known, the likelihood of three negative C-PCRs in the combination of a negative C-Ag is highly unlikely [3-4].

Aspiration pneumonitis and fat embolism remained in our differential as we continued to care for the patient [5]. The metabolic theory for fat embolism focuses on the convalesce of chylomicrons due to inflammatory response [6]. Fat embolism syndrome findings include a presentation of 24-72 h of insult characterized by hypoxia, central nervous system depression, pulmonary edema, and a petechial rash. Of these, our patient did not have a petechial rash or central nervous system depression [6-8]. Our patient also had a long bone fracture which has the highest incidence of fat embolism syndrome among orthopedic fractures [9]. The computed tomography angiography (CTA) findings discussed above also are consistent with fat embolism syndrome. Using Schonfeld's criteria (Table 1), the patient had 11 points with only five being required for fat embolism diagnosis. After proning and airway pressure release ventilation trial the patient had rapid improvement of his hypoxic condition which is more consistent with fat embolism syndrome.

**Table 1:** Schonfeld's Criteria (total score > 5 required for diagnosis).

Criteria	Points
Petechiea	5
Chest X-ray change (diffuse alveolar change)	4
Hypoxemia (PaO2)	3
Fever (temperature > 38°C)	1
Tachycardia (HR > 120 bpm)	1
Tachypnea (>30/min)	1
Confusion	1

Although our CT findings could suggest aspiration pneumonia, the patient had bilateral consolidations and ground glass opacities and did not have a witnessed aspiration event or any history of swallowing difficulty.

## Conclusions

Fat embolism is a strong possibility, especially after a long bone fracture, and, should be vigilantly monitored. It may mimic COVID-19 but as the guidance for COVID-19 treatment fluctuates as we learn more about the disease, the treatment for fat embolism for mechanically ventilated regimen includes high positive end-expiratory pressure (PEEP) similar to acute respiratory distress syndrome (ARDS). However, differences in the course of treatment will show a rapid improvement in oxygenation status for fat embolism syndrome with ventilatory support whereas severe COVID-19 pneumonia will often have a protracted course. These differences can help distinguish the causes for ARDS in patients with long bone fractures in the COVID-19 era. 
